# Automatic identification method of bridge structure damage area based on digital image

**DOI:** 10.1038/s41598-023-39740-z

**Published:** 2023-08-02

**Authors:** Jinchao Wang, Houcheng Liu, Zengqiang Han, Yiteng Wang

**Affiliations:** 1 Institute of Rock and Soil Mechanics, Chinese Academy of Sciences, Wuhan, 430071 Hubei China; 2 State Key Laboratory of Geomechanics and Geotechnical Engineering, Wuhan, 430071 Hubei China; 3 Wuhan Zhongke Kechuang Engineering Testing Co., Ltd., Wuhan, 430071 Hubei China

**Keywords:** Microbiology, Molecular biology, Diseases, Pathogenesis

## Abstract

It is of great scientific and practical value to use effective technical means to monitor and warn the structural damage of bridges in real time and for a long time. Traditional image recognition network models are often limited by the lack of on-site images. In order to solve the problem of automatic recognition and parameter acquisition in digital images of bridge structures in the absence of data information, this paper proposes an automatic identification method for bridge structure damage areas based on digital images, which effectively achieves contour carving and quantitative characterization of bridge structure damage areas. Firstly, the digital image features of the bridge structure damage area are defined. By making full use of the feature that the pixel value of the damaged area is obviously different from that of the surrounding image, an image pre-processing method of the structure damaged area that can effectively improve the quality of the field shot image is proposed. Then, an improved Ostu method is proposed to organically fuse the global and local threshold features of the image to achieve the damaged area contour carving of the bridge structure surface image. The scale of damage area, the proportion of damage area and the calculation rule of damage area orientation are constructed. The key inspection and characteristic parameter diagnosis of bridge structure damage area are realized. Finally, test and analysis are carried out in combination with an actual project case. The results show that the method proposed in this paper is feasible and stable, which can improve the damage area measurement accuracy of the current bridge structure. The method can provide more data support for the detection and maintenance of the bridge structure.

## Introduction

The bridge plays a very important leading role in the national economic development, which can bring huge economic and social benefits to the country, so the country pays great attention to the safe use of bridge^1^. However, due to the erosion of natural environment, the aging of building materials, the fatigue effect of bridge itself and other adverse factors, the resistance of bridge will inevitably decline, which will lead to the decline of ability to resist natural disasters and even the normal environment. In extreme cases, it can cause catastrophic accidents^2^. Bridge structure is an important part of a country's infrastructure construction. Bridge makes frequent communication between different regions possible. However, under the influence of multiple environmental factors, with the growth of service time, bridge structure will appear different degrees of structural damage. Bridge structure is different from the general public buildings. If the bridge collapses or the lack of safety in use, it will bring great harm to our country and people bring huge losses, so the bridge structure needs to maintain a high safety state, and the frequency of maintenance is also higher than that of general public buildings^3^. In order to ensure the applicability, safety and durability of bridge under normal operation state, it is necessary to adopt effective technical means to carry out real-time and long-term monitoring and early warning for the structural damage of bridge, so as to provide scientific basis for the safety assessment of bridge structure^4–6^.

With the completion of many expressways, the bridge structure is becoming larger and larger. The use of viaduct, continuous beam bridge and continuous rigid frame bridge makes the inspection of bridge become a time-consuming and laborious work. In the maintenance of concrete bridges, damage area detection is one of the important inspection and diagnostic steps for bridge structures. Usually, bridge surface cracks can be used to evaluate the bearing capacity, water tightness and service life of concrete structures. In the static load bending test of bridge, the detection of cracks at the bottom of beam is completed manually. Artificial vision detection depends on the operator's experience and skill level, which is time-consuming and low safety, so it is difficult to evaluate the detection effect objectively. Automatic detection of damage area in bridge surface image is very effective in nondestructive testing^7^. Regular detection of beam cracks can also be used to evaluate the safety and reliability of concrete bridge structure and prevent accidents. Traditional manual detection is inefficient and affects the smooth flow of road traffic. It is a real-time, non-destructive, high-precision and low-cost detection method to find the diseases on the bottom plate of bridge superstructure by using image processing technology to analyze the pictures of bridge structure. At present, there are complete sets of equipment using this method or principle in other fields, but these equipment are mostly developed abroad and expensive. In addition, due to the diversity and uncertainty of bridge diseases, the algorithm in the process of disease detection still needs further research.

Digital image processing technology originates from human vision, it is a comprehensive discipline, involving optics, electronics, photography, computer technology and other fields^8–10^. In recent years, due to the continuous decline of price of computer equipment, the digitization of digital image and the increasing popularity of display equipment, more and more fields use the method of computer image processing for detection, from the traditional manufacturing industry to the field of microelectronic integrated chip. It is the application of computer graphics in the field of advanced production from the measurement of product flatness and size to the detection of bad information such as product defects and cracks. The role of digital image processing technology in the national economy is more and more obvious. From the perspective of development, we can see that digital image processing technology will play an increasingly important role in the future development. With the development of image acquisition and recognition technology, the high-definition camera is used to take photos of bridge to be detected area, and the image processing algorithm is used to process the image, so as to extract features such as cracks, etc., which has become a new method for automatic identification of bridge damage area in bridge static load bending test^11^. Computer vision and artificial intelligence technology have made significant progress in the automatic recognition of damaged areas, which can improve the image recognition energy of damaged areas^12–15^. However, in data training, traditional manual intervention can easily reduce recognition efficiency and accuracy, and it is necessary to improve the training efficiency and accuracy of data samples. Traditional image recognition network models are often limited by the lack of on-site images.Therefore, this paper adopts digital image processing technology based on optical measurement method to collect, analyze and process bridge structure diseases, which proposes an automatic identification method of bridge structure damage area based on digital image, so as to realize artificial intelligence measurement of bridge structure damage area.

## Image features of bridge structure damage area

**Figure 1:**
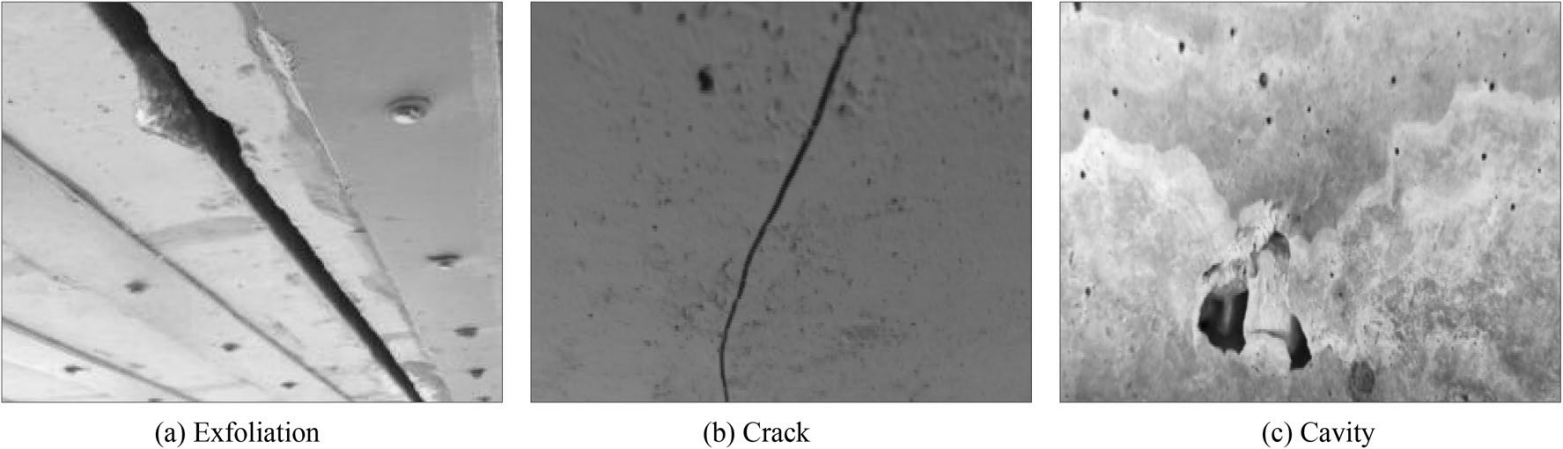
Surface damage characteristics of typical bridge structure.

In the service process of bridge, the load effect, fatigue and cavity effect, material aging and other factors will inevitably cause damage in some areas of bridge structure. If the damage of bridge structure can not be detected and repaired in time, it will seriously affect the safety of bridge structure. The appearance damage characteristics of bridges are mainly cracks and cavities. At present, the main technical means to obtain the appearance damage characteristics of bridge structure is digital image measurement technology. Through digital image acquisition, combined with image processing and pattern recognition and classification technology, the crack and cavity characteristics of bridge structure can be obtained. The artificial observation of damaged area of bridge usually depends on the color difference and experience between the damaged area and the surrounding environment. Due to the limited shooting environment, the bridge structure images are often not parallel to the camera's imaging plane. At this time, the structure images with perspective projection deformation are obtained, and the angle of view of damaged area needs to be corrected. Typical damage characteristics of bridge structure are shown in Fig. 1. In the follow-up analysis and processing, through the use of MATLAB and VC +  + software combined with the method described in this paper, the images taken from camera are processed by digital image processing.

From the visual characteristics, in Fig. 1a, there is a phenomenon of block falling around the tension joint of bridge structure. By comparing the width of tension joint, it can be seen that the width of tension joint is not consistent up and down, which is mainly due to the shooting angle and the bridge structure detection area is not vertical. There is a certain shooting angle between camera and bridge structure. There is a significant difference between the pixel value of block dropping area and that of surrounding image. In Fig. 1b, there are cracks on the surface of bridge structure, and the cracks usually appear as continuous slender damage areas. There is a significant difference between the pixel value of crack area and that of surrounding image. The pixel value of center of crack is the smallest, and gradually increases from the center of crack to the area without cracks on both sides. In Fig. 1c, there are cavities on the surface of bridge structure, which are usually discontinuous spherical damage areas. There is a significant difference between the pixel value of hole area and that of surrounding image.

**Figure 2:**
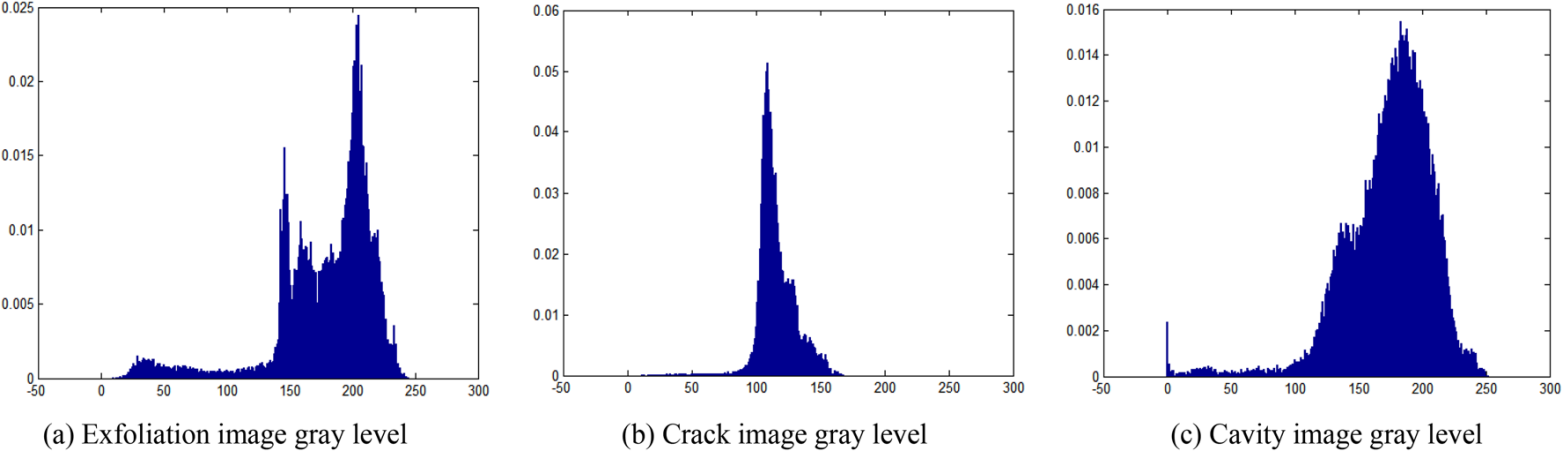
Gray distribution of typical bridge structure surface damage characteristics.

The damage area in the image collected by the camera is automatically identified according to the pixel value of each pixel, whose value ranges from 0 to 255. The gray value distribution of image in Fig. 1 is shown in Fig. 2. Figure 2 is a histogram of pixel gray value and gray value proportion. The x axis in Fig. 2 represents the pixel gray value. The y axis in Fig. 2 represents the proportion of gray value. Figure 2a corresponds to the image feature of Fig. 1a. Figure 2b corresponds to the image feature of Fig. 1b. Figure 2c corresponds to the image features of Fig. 1c. From the difference characteristics of pixel values, in Fig. 2a) the gray values corresponding to the block dropping image are mainly concentrated in 150—210, and the gray values have multiple peaks, and the gray values are relatively scattered. In Fig. 2b, the gray value of crack image is mainly concentrated in 100–130, and there is a single peak value in the gray value, and the gray value is relatively concentrated. In Fig. 2c, the gray value of hole image is mainly concentrated in 130–220. The gray value has a single peak. The gray value is relatively scattered.

## Image preprocessing of structural damage area

**Figure 3:**
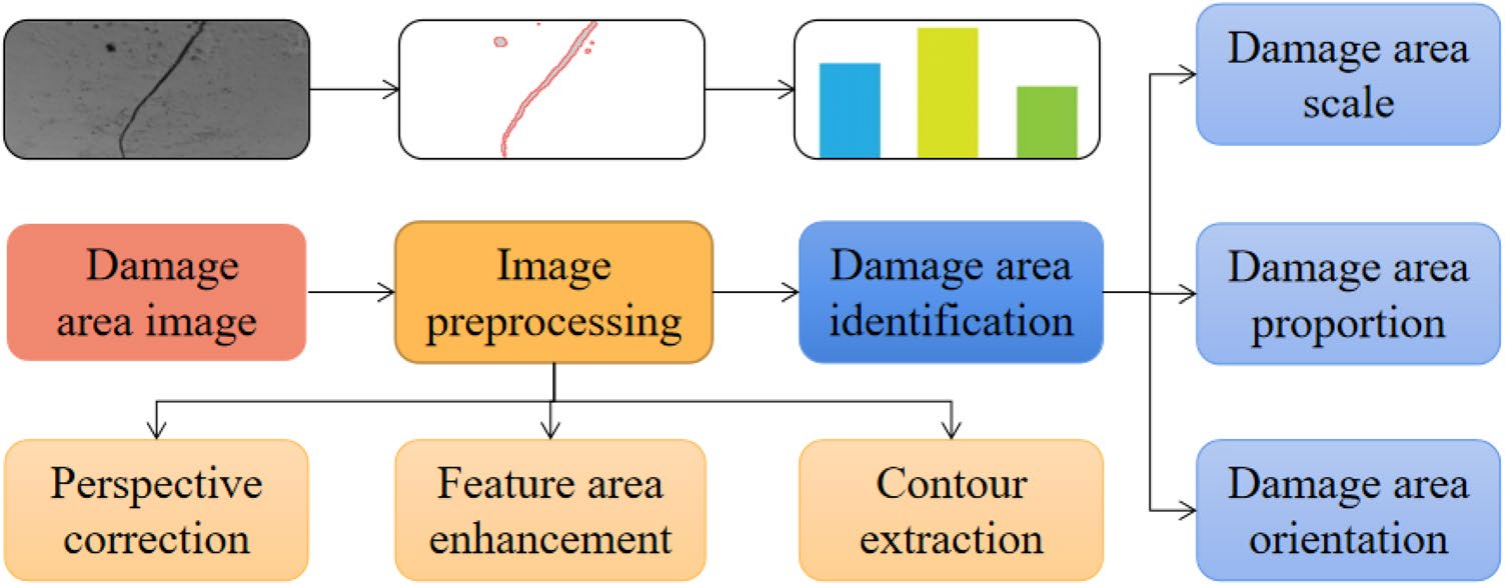
Technical route schematic diagram.

The technical route of this method is shown in Fig. 3. The image of the original damage area is preprocessed, and then feature parameters are extracted. The most important part is the image preprocessing stage. The results of the processing affect the recognition effect of the overall structural parameters. The image quality of bridge structure surface depends on the image quality of digital camera and the on-site photography conditions. In order to facilitate the subsequent image processing, it is necessary to correct the view angle of damage area, enhance the damage feature area and extract the contour of damage area according to the image characteristics of bridge structure damage area.

### View angle correction of damaged area

**Figure 4:**
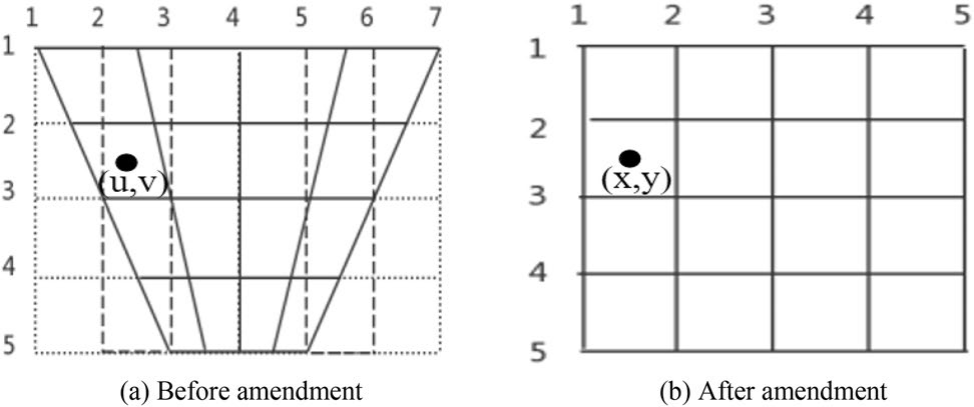
Schematic diagram of perspective projection deformation.

The plane of bridge structure damage area photographed on site is often not parallel to the imaging plane of camera. At this time, the crack image with perspective projection deformation is obtained. The bridge structure damage area will produce "near wide and far narrow" deformation, which increases with the increase of shooting angle^16^. In order to obtain the accurate image of bridge structure damage area, the perspective of image needs to be modified by perspective projection transformation. According to the spatial model of digital image, the coordinates of image points are the result of perspective lens in the imaging plane. The schematic diagram of perspective projection deformation is shown in Fig. 4.

The intersection of lines is the row and column position of pixels. The coordinates of pixels obtained by perspective projection deformation are not all integers. There are blank areas in the result image, so it is necessary to calculate the pixel values of integer coordinates by interpolation fitting. Suppose that the coordinates of a point in the surface image of bridge structure before correction are (u, v), and the coordinates of point in the image after correction are (x, y). According to the principle of perspective projection imaging, the corresponding coordinates in the surface image of bridge structure before and after the transformation meet the following relationship^17^:

1 $$ k\left[ \begin{gathered} x \hfill \\ y \hfill \\ 1 \hfill \\ \end{gathered} \right] = \left[ \begin{gathered} a_{1} \;\;a_{2} \;\;a_{3} \hfill \\ a_{4} \;\;a_{5} \;\;a_{6} \hfill \\ a_{7} \;\;a_{8} \;\;a_{9} \hfill \\ \end{gathered} \right]\left[ \begin{gathered} u \hfill \\ v \hfill \\ 1 \hfill \\ \end{gathered} \right] $$

Among them: elements a_1_, a_2_, a_4_ and a_5_ are the image linear transformation coefficients, which can make the image produce rotation, shearing and scaling deformation. a_3_ and a_6_ are the translation coefficients, which can make the image produce rigid body displacement. a_7_ and a_8_ are the perspective projection transformation coefficients, which control the perspective projection deformation of image. *k* is the variable homogeneous coordinate coefficient. *k* is a non-zero real number. If *k* = 0, because the divisor cannot be 0, (x, y, 0) is a point at infinity, and there is no two-dimensional point corresponding to (x, y, 0). Usually, selecting *k* = 1 can simplify the coordinates to triples (x, y, 1) and improve transformation efficiency. If the coefficients a_1_ ~ a_9_ are multiplied by any non-zero real number at the same time, the corresponding relationship between (u, v) and (x, y) will not be changed. Therefore, in order to simplify without affecting other parameter changes, it is advisable to take a_9_ = 1, and the following equations can be obtained^17^.

2 $$ \left\{ \begin{gathered} a_{1} u + a_{2} v + a_{3} - a_{7} ux - a_{8} vx = x \hfill \\ a_{4} u + a_{5} v + a_{6} - a_{7} uy - a_{8} vy = y \hfill \\ \end{gathered} \right. $$

**Figure 5:**
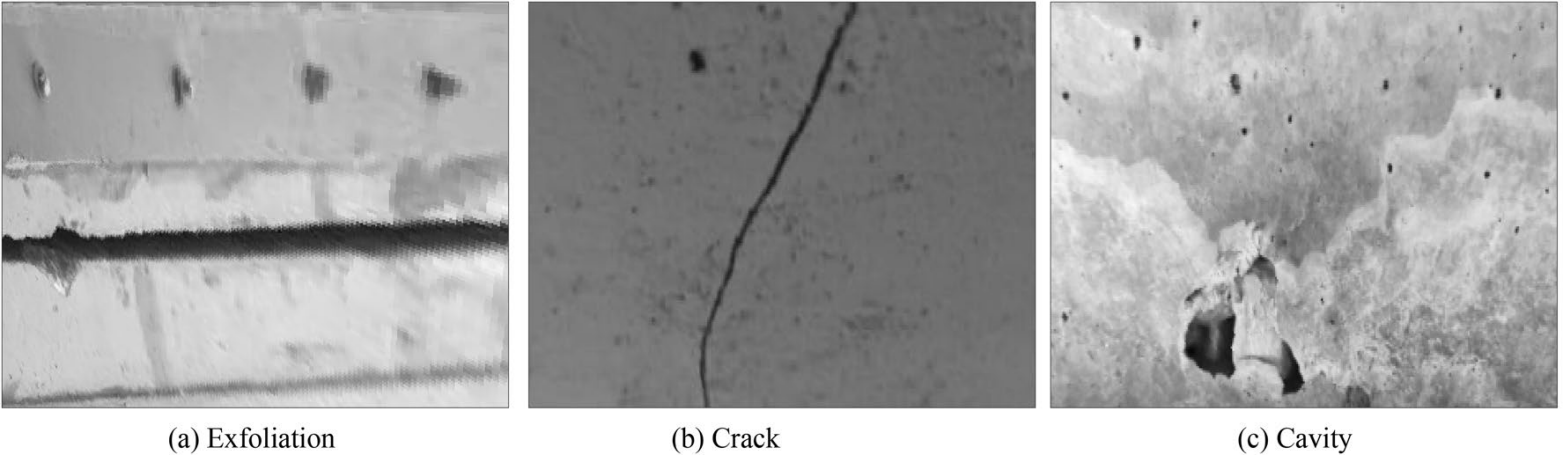
Damaged area image after view angle correction.

When the image coordinates before and after the transformation of four non collinear points are known, there are eight unknowns and eight equations, and the transformation matrix coefficients can be solved. Then, the coordinates of each pixel of image after perspective projection deformation are solved by the relation (1). By performing the above processing on the three images in Fig. 1, the angle correction processing of different damage areas is completed, and the results are shown in Fig. 5. It can be seen from the Fig. 5 that the image after the view angle correction of damage area can better reflect the real characteristics of damage area, which is convenient for the subsequent image automatic recognition and parameter extraction.

### Image feature area enhancement

**Figure 6:**
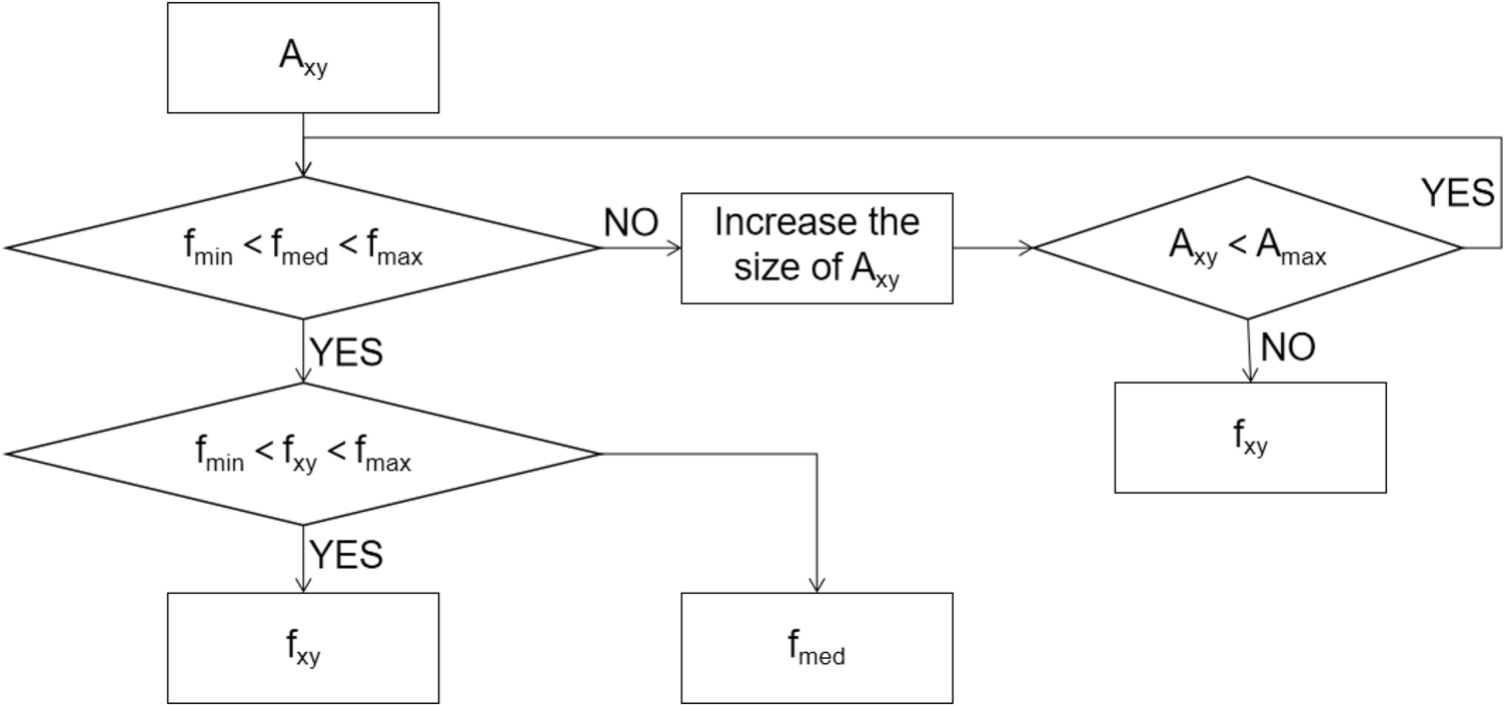
Flow diagram of algorithm basic steps.

In order to facilitate the subsequent image processing, image denoising should be carried out. Based on the collected image, the image denoising processing can be carried out. The image noise processing can use the image decomposition and reconstruction method of two-dimensional wavelet analysis to eliminate the influence of noise. Image denoising technology is mainly aimed at removing the interference of random signals in the process of image acquisition or transmission. In the radiation environment, a large number of impulse noises lead to image degradation. Impulse noise is also called salt and pepper noise^18^. The probability density function is constructed to judge whether the image feature area is salt and pepper noise. Its probability density function is expressed as follows:

3 $$ p(z) = \left\{ \begin{gathered} p_{a} \;\;\;\;\;\;\;\;z = a \hfill \\ p_{b} \;\;\;\;\;\;\;\;z = b \hfill \\ 0\;\;\;\;\;\;\;\;\;\;others \hfill \\ \end{gathered} \right. $$

when b > a, the gray value b is bright in the image, that is salt noise, and the gray value a is dark, which is pepper noise. In view of image noise existing in the bridge structure damage area, adaptive median filter can effectively retain the image edge features, so this paper selects adaptive median filter method for image denoising. The principle of adaptive median filtering method is to adjust the size of filtering window by the noise density, and use different processing methods to process the noise points and signal points. Then, median filter the noise points to keep the gray value of signal points unchanged. The gray value of pixel (x, y) in the bridge structure image is f_xy_, the current working window is represented by A_xy_. The preset maximum allowable window is represented by A. the minimum, median and maximum gray values in A are f_min_, f_med_ and f_max_ respectively. The basic steps of algorithm are shown in Fig. 6.

**Figure 7:**
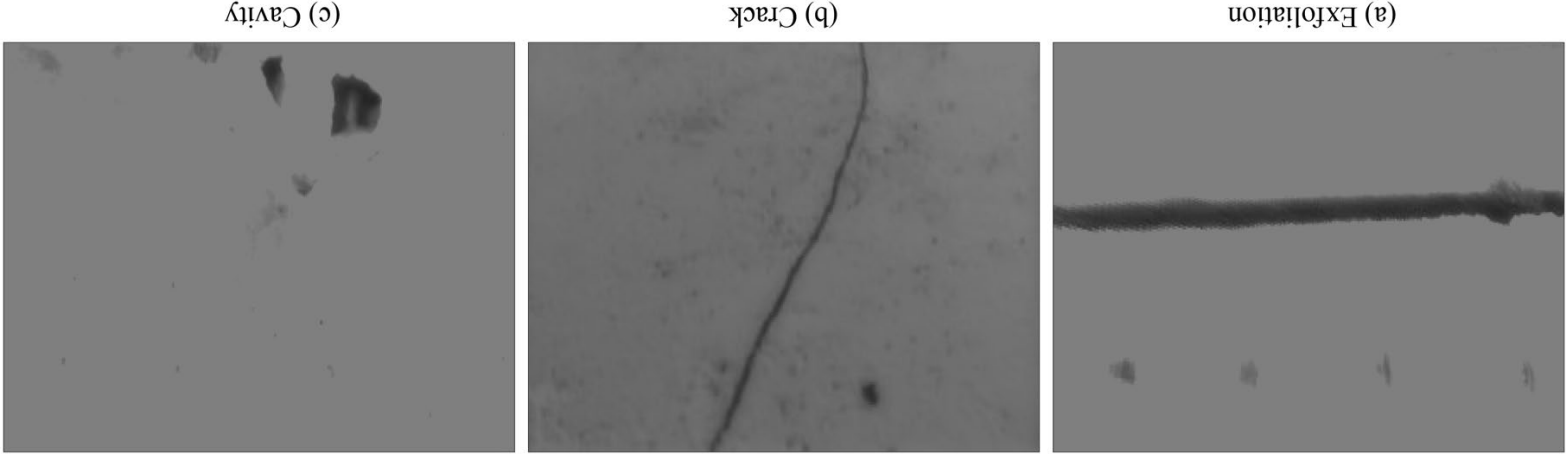
Part of enhanced image.

In the adaptive median filtering algorithm, the detection and identification of noise points are based on f_min_ and f_max_. When f_min_ < f_med_ < f_max_, f_med_ is not noise, so f_min_ < f_xy_ < f_max_ is used to judge whether f_xy_ is noise. If f_xy_ and f_med_ are not impulse noise, f_xy_ is given priority. In order to eliminate the interference caused by surface stains, gradual bumps and potholes in the image, it is necessary to enhance the image feature area and highlight the feature information of damaged area after completing the adaptive median filtering of image. In order to reduce the interference of external non important information to the image, this paper uses the average method of high-frequency component extraction. The reference image is obtained by Gaussian low-pass filtering, and then the original image and the reference image are averaged to obtain the absolute value of pixel value of image region, which can realize the enhancement of image feature region. The partially enhanced image is shown in Fig. 7.

### Contour extraction of damaged area

**Figure 8:**
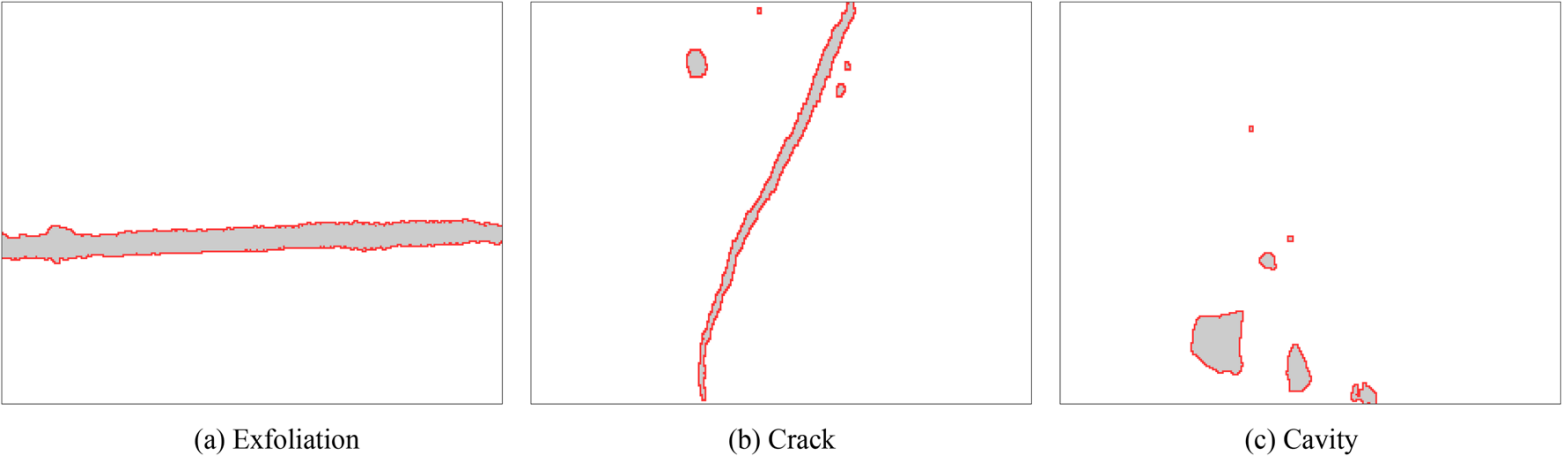
Damaged area image after contour extraction.

The segmentation of bridge damage area is a process of binarization of bridge structure surface image. The process of binarization is to segment the image by thresholding. According to different threshold selection methods, the main image segmentation algorithms are histogram threshold method, iterative method and Otsu method^19,20^. Due to the complexity of the scene shooting environment of the bridge, the gray value of some background parts of the damaged area is relatively close to the gray value of the feature area. The diversity of the features of the damaged area feature and the gray change makes the traditional binary method difficult to work, resulting in the processing effect is not very reasonable, which is easy to affect the subsequent processing of the image of the damaged area. Therefore, this paper proposes an improved Otsu method to extract the contour of bridge damage area image. Aiming at the color gradient difference between the damaged and non damaged regions of bridge, an improved Otsu method is proposed by fusing the global and local threshold features of image. Assuming that the bridge damage area image to be identified and analyzed is F, and the number of pixels is M × N matrix, the concrete steps of bridge damage area segmentation are as follows:

1. According to the characteristics of color gradient between the bridge damage area and the non bridge damage area, the image F of bridge damage area after de-noising is gradient processed and binarized to form the gradient image F1. Based on the denoised image f of bridge damage area, the global threshold method is used to segment the image to obtain the initial binary image F2.
2. The image of bridge damage area is divided into m × n regions, and the pixel matrix of each element region is (M / m) × (N / n). The Otsu method is used for image segmentation of each element region. The m × n element images are reconstructed according to the division order, and the reconstructed image f_i_ is formed by splicing, where m is the common divisor of M and n is the common divisor of N. The larger the value of m and n are, the smaller each unit area of bridge damage area image is, and the more obvious the distinction of bridge damage area is. But the smaller each unit area of bridge damage area image is, the more obvious the stitching trace of reconstructed image f_i_ is, which should not be too large. Therefore, it is necessary to select the appropriate m and n according to the size of bridge damage area to ensure that each unit area can cover the minimum size of damage area. m and n are usually 4 or 8. The reconstructed image formed by the unit area f_i_ which can cover the smallest size of damaged area is called F3.
3. Because the local threshold processing is used in the reconstructed image, the bridge damage area can be effectively distinguished from the surrounding environment. When processing the damaged area in the window, individual noise will cause the threshold mutation, which is easy to appear in the background area of artifacts. At this time, the intersection of images F1, F2 and F3 is taken to obtain the segmentation image F4 of bridge damage area. The image F4 not only uses the gradient difference of damage area, but also combines the advantages of global threshold method and the local threshold method, which can effectively distinguish the bridge damage area and remove the artifacts caused by the local threshold. The processed image is shown in Fig. 8.

## Characteristic parameters extraction of structural damage 

In the automatic identification of bridge structure damage area, the scale parameter, proportion parameter and orientation parameter of damage area are important inspection and diagnosis parameters of bridge structure. Therefore, this section mainly carries out analysis and research from the extraction of damage area scale, proportion and orientation parameter.

### Scale characteristics of damage area

In the image acquisition of bridge structure damage area, it is necessary to directly observe the structure damage information by zooming. Before labeling the structural damage information, the first step is to calibrate the size of structural damage image. The size of each pixel can be obtained by the size calibration, so that the actual size can be obtained directly through the calculation of pixel in the later structural damage identification and crack measurement. In structural damage calibration, the bridge structural feature size or easily identified location is usually selected for calibration, and the bridge structural dimension Q can be obtained from the bridge design data.

The distance between camera and bridge may be different when taking the damaged area image of bridge structure. The closer the distance between camera and bridge, the higher the image quality of bridge structure damaged area. But in a certain range between camera and bridge, the images can be effectively recognized. In order to eliminate the difference influence of effective shooting distance on the damage area scale calculation, it is necessary to modify the image according to the distance between camera and bridge. The number of width pixels P can be obtained by selecting the mean value of multiple different positions with the same design size from the real image, and the actual size C represented by each pixel can be obtained by this method. Considering the scaling and deformation operation of image during actual observation, the actual scaling ratio value a needs to be added to obtain the scaled pixel size value C_x_.

4 $$ C_{x} = \frac{Q \cdot a}{P} $$

When obtaining the structural damage information, we only need to get the relative position of damage area from the calibration pixel on the image to get the location of structural damage in the bridge. The scale characteristics of damage area mainly include the maximum continuous length and the maximum radius of damage area. In order to obtain the maximum continuous length of damage area, the contour continuous tracking method is used to calculate the length. The number of pixels N_l_ between the farthest two points on the outer contour of damage area is calculated statistically. The maximum continuous length of e-th area in the damage image can be expressed as L(e).

5 $$ L(e) = N_{l} \cdot C_{x} $$

In order to obtain the maximum radius of damage area, the maximum circle search method is used to search the radius. The central axis node a of damage area is determined automatically or manually. The central axis node a of damage area is taken as the center of circle, and its coordinates are expressed by (i, j). Make a search circle with radius r = 1. If the arc boundary of search circle does not touch the pixels in the damaged area, increase the radius of search circle, that is, r = r + Δr. By increasing the radius of search circle until the boundary of arc contacts the pixels of damage area, the search circle is the maximum inscribed circle of damage area. If the number of pixels of maximum inscribed circle radius of damaged area is N_r_, the radius of damaged area is expressed by $R(i,j)$. The expression is as follows:

6 $$ R(i,j) = \frac{{2N_{r} \cdot C_{x} }}{\pi } $$

### Proportion characteristics of damage area

In order to identify and extract the characteristic parameters of damage area, it is necessary to mark each damage area to obtain the number of damage areas. The number of damage areas is defined as the number of closed areas with a certain trap area in the bridge structure surface image after the damage area contour is filled, and the number of damage areas in the bridge structure surface image is recorded as E. In this paper, the number marking algorithm based on equivalent pairs is used to obtain the number of damaged areas *E*. On the basis of traditional eight connected domain marking algorithm^21^, the equivalence relationship between the temporary marking and the final marking is established and stored in the linked list structure to improve the calculation speed and accuracy. The specific steps mainly include: image preliminary marking, sorting the equivalence list and image substitution.

Firstly, each pixel is given a temporary label, and the equivalence relation of temporary label is recorded in the equivalence table. Every pixel in the image is traversed. If it is the target pixel, eight neighborhoods are searched. The search follows the rules of left, top left, top right. If the neighborhood is not marked, a new tag is assigned. Otherwise, the current tag is assigned as a neighborhood tag, and the equivalence between the two is recorded. Then, all the temporary markers with equivalence relation are equivalent to the minimum value. The damage areas are relabeled in the order of natural numbers to obtain the equivalence relation between the temporary markers and the final markers. Finally, the image is traversed again, and the temporary mark is replaced by the final mark. After these steps, the damage areas in the image are marked with continuous natural numbers from top to bottom and from left to right. The natural number of final mark is the number of damage areas E in the bridge surface image.

Assuming that the pixel value of damaged area is 0, the damaged area is divided into grids. Each grid row and each grid column contains several points in the damaged area. In each column, the leftmost point is the left boundary point, and the rightmost point is the right boundary point. If there is only one point in each grid line, it is not only the left boundary point, but also the right boundary point. The area of damage area is the sum of pixels contained in the corresponding damage area grid. The damage area is divided into n × n meshes. The left most point of each row is P(x_i_, y_ij_), and the right most point is Q(x_i_, y_ij_'). Then the pixels contained in the damaged area are y_ij_-y_ij_' + 1. Therefore, the total area $S(E)$ of damage area is:

7 $$ S(E) = \frac{{1}}{{2}} \cdot C_{x} \cdot C_{x} \cdot \sum\limits_{i = 1}^{n} {(y_{ij} - y_{ij}{\prime} + 1)} $$

According to the relation (7), when calculating the area of a damaged area, the pixel coordinates of leftmost and rightmost sides in each row of grid can be calculated separately, instead of considering the corresponding relationship of leftmost and leftmost points each time. Therefore, the most critical step of area calculation is to determine the type of boundary points, which can be determined by the in and out chain code of contour. According to the clockwise contour, the left and right boundary determination table is set.

### Orientation characteristics of damage area

In order to get the orientation features of each point in the bridge structure damage area image, we need to get the gradient values of pixels in the x and y directions. Sobel operator method is used to obtain the gradient values $\partial_{x} (u,v)$ and $\partial_{y} (u,v)$. In order to make the orientation information of each pixel as accurate as possible, the local area pixels are used as reference to calculate. In a pixel I(i, j), a rectangular region with height h and width w is selected as the center, and the average values $V_{x} (i,j)$ and $V_{y} (i,j)$ of gradient values in x and y directions of pixel are calculated.

8 $$ \left\{ \begin{gathered} V_{x} (i,j) = \sum\limits_{{u = i - \frac{w}{2}}}^{{i + \frac{w}{2}}} {\sum\limits_{{v = j - \frac{h}{2}}}^{{j + \frac{h}{2}}} {2\partial_{x} (u,v)\partial_{y} (u,v)} } \hfill \\ V_{y} (i,j) = \sum\limits_{{u = i - \frac{w}{2}}}^{{i + \frac{w}{2}}} {\sum\limits_{{v = j - \frac{h}{2}}}^{{j + \frac{h}{2}}} {2\partial_{{_{x} }}^{2} (u,v) - \partial_{{_{y} }}^{2} (u,v)} } \hfill \\ \end{gathered} \right. $$

where, (i, j) is the center of rectangular block. u, v are the independent variables along the x, y direction respectively. The expression of phase $\theta (i,j)$ at each point is as follows:

9 $$ \theta (i,j) = \frac{1}{2}\arctan \frac{{V_{y} (i,j)}}{{V_{x} (i,j)}} $$

The spatial direction of $\theta (i,j)$ is orthogonal to the main direction of Fourier spectrum in the selected h × w window. Due to the noise and impurities on the bridge surface, the direction of calculation may be deviated. Gaussian low-pass filter is used to reduce the influence, that is, the weighted average of direction in the neighborhood of a pixel is used to replace the direction of pixel. The orientation of damaged area is the cumulative average of orientation of pixels in each local area. If the orientation of e-th damaged area is represented by S_r_(e), and the number of pixels with 0 pixel in the e-th damaged area is represented by e_k_, then the expression of S_r_(e) is as follows:

10 $$ S_{r} (e) = \frac{1}{{{\text{e}}_{k} }}\sum\limits_{i = 1}^{n} {\sum\limits_{j = 1}^{n} {\theta_{e} (i,j)} } $$

## Test analysis

### Data acquisition

Taking the Huanshui River Bridge in Hubei Province of China as an example, the bridge is located in the northwest of Hubei Province of China, where the river channel is straight during flood period. The skew angle between the bridge axis and flood flow is 5 degrees. By consulting the geological materials of various surveys before bridge construction, it can be seen that the geology of bridge site is relatively stable on the whole. Some parameters are shown in Tab. 1.

**Table 1:** Part of parameters.

Riverbed area	Main types	Thickness
Surface layer of riverbed	Clay layer, loam layer	0 to 9 m
Middle layer of riverbed	Fine sand layer, coarse sand layer	2 to 9 m
Lower layer of riverbed	Gravel layer	3.5 to 9 m
	Cobble layer	1.3 to 9 m
	Pebble layer	N/A

**Figure 9:**
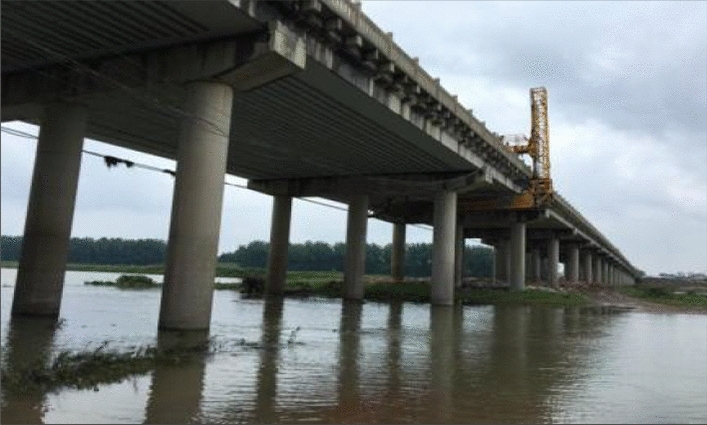
Physical image of bridge.

The bottom elevation is -10.5 ~ -21.3 m. The river reach of bridge site is considered as grade 6 navigation requirements. Longitudinal slope is set in the longitudinal plane of bridge axis, vertical curve is set near the pile number in the center of bridge. The plane of bridge axis is a straight line. There are five pairs of piers in the whole bridge. The span is 3 × (5 × 20) + 2 × (4 × 20) m, and the total length of bridge is 466.54 m. The superstructure of bridge adopts prestressed concrete hollow slab with continuous deck. The substructure is a column pier with a diameter of 1.2 m, and the foundation is a bored pile with a diameter of 1.4 m. The bored pile is designed according to the requirements of friction pile. The physical image of the bridge captured on site using a mobile phone is shown in Fig. 9.

**Figure 10:**
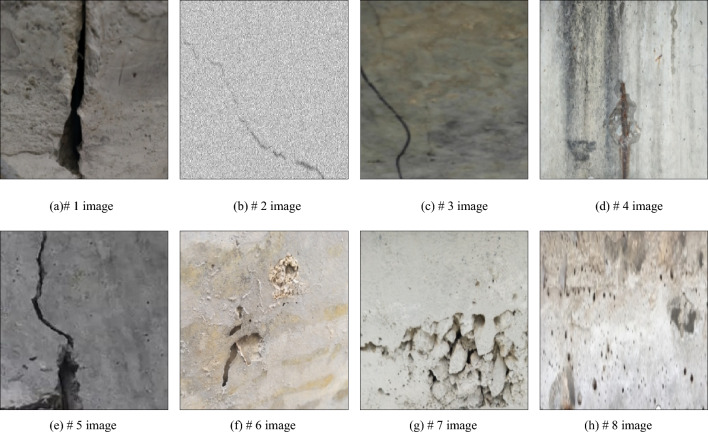
Part of original image.

In order to ensure the safe operation of bridge, through the technical condition evaluation and bearing capacity inspection and evaluation of bridge, evaluate the technical condition and bearing capacity of bridge, judge its safety and applicability, provide the exact technical basis for the upgrading and reinforcement of bridge, and provide the original accumulation for the establishment of bridge health files and dynamic management of maintenance data. For this purpose, the digital camera is used to collect images of 8 damaged parts of bridge. The original image results are shown in Fig. 10. The actual size of each image is 1 m × 1 m.

### Data processing

**Figure 11:**
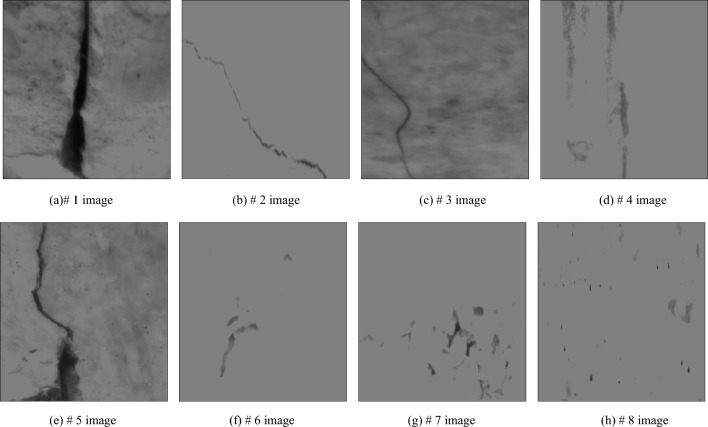
Image after image processing.

By analyzing the image features of bridge structure damaged area, the view angle of damaged area is modified. The noise of damaged image is removed, and the contour of damaged area is extracted. After the damage area view angle correction processing and the damage image denoising processing for the bridge structure surface image, the 8 images obtained are respectively as shown in Fig. 11. It can be seen from Fig. 10 that there are the following problems in some structures of bridge: Cracks in the main beam, some of which are close to or exceed the specified limit value; honeycomb, pockmarked surface, spalling and damage of concrete; corrosion of local reinforced steel plate and damage of waterproof coating.

**Figure 12:**
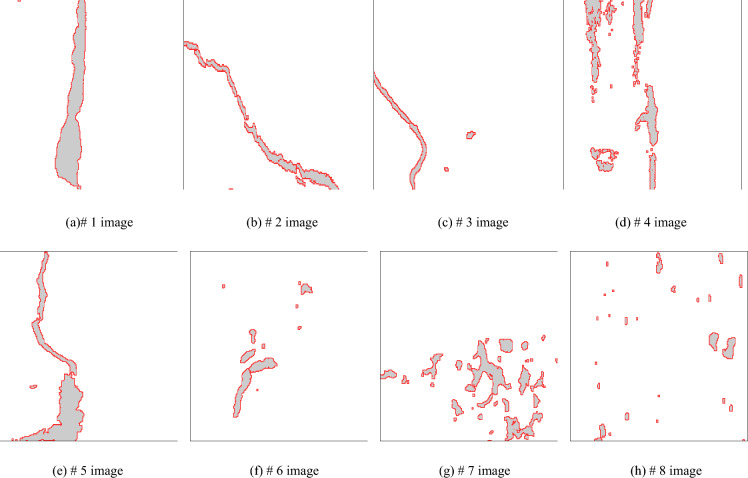
Extracted image of damage area.

After the damage area contour extraction, the obtained damage area is shown in Fig. 12. On the basis of obtained damage area image, through the automatic identification method of bridge structure damage area, the calculation of damage area scale, damage area proportion and damage area orientation is realized. The actual size of each pixel in the image is 4 mm × 4 mm. The number of damaged areas E in different bridge structure images is calculated and counted respectively. The number of damaged areas in different bridge structure images is different. By combining Eq. (5) in Section "Characteristic parameters extraction of structural damage " above, the length L of the damaged area can be calculated. By combining Eq. (6) in Section "Characteristic parameters extraction of structural damage " above, the radius R of the damaged area can be calculated. By combining Eq. (7) in section "Characteristic parameters extraction of structural damage " above, the area S of the damaged area can be calculated. By combining Eqs. (8) - (10) in Section "Characteristic parameters extraction of structural damage " above, the orientation Sr of the damaged area can be calculated. In order to facilitate statistical analysis, the feature parameter values of largest damage area in the bridge structure image are listed in Tab. 2. The length, radius, area and orientation of largest damaged area in the bridge structure image are respectively expressed by L(max), R (max), S(max) and S_r_(max). The specific values are shown in Tab. 2.

**Table 2:** Part of calculated data.

*E*	#1 image	#2 image	#3 image	#4 image	#5 image	#6 image	#7 image	#8 image
	1	3	3	22	3	11	33	25
L (max)	1.000 m	0.682 m	0.911 m	0.417 m	0.753 m	0.277 m	0.264 m	0.134 m
R (max)	0.138 m	0.067 m	0.035 m	0.583 m	0.117 m	0.517 m	0.067 m	0.053 m
S (max)	0.073m^2^	0.034m^2^	0.009m^2^	0.119m^2^	0.056m^2^	0.019m^2^	0.053m^2^	0.022m^2^
S_r_ (max)	3.120°	152.103°	160.457°	171.335°	5.642°	8.412°	143.543°	32.141°

### Result analysis

**Figure 13:**
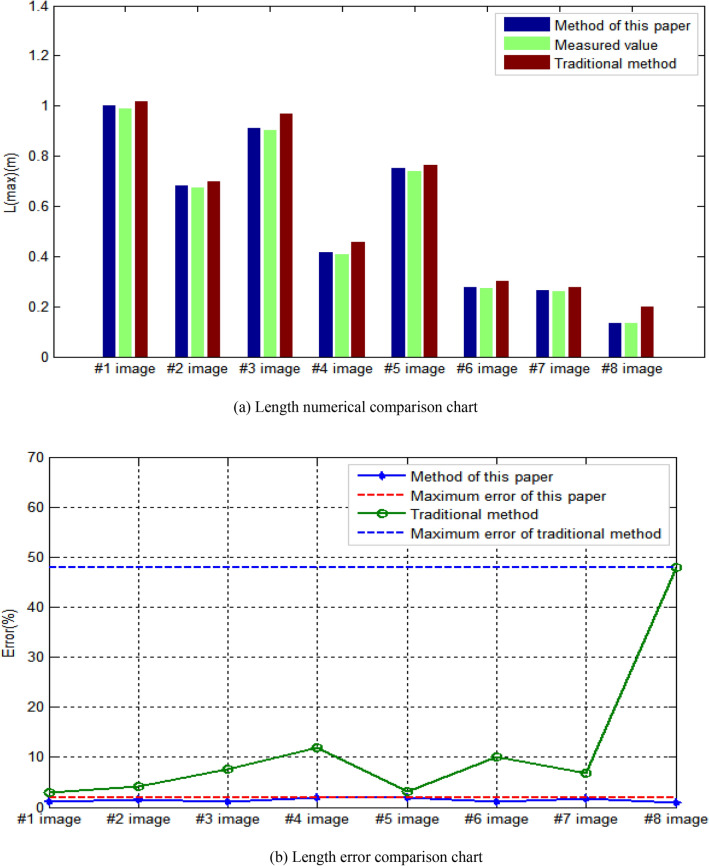
Comparison of length value and error.

In order to verify the feasibility and accuracy of method, the method is compared with the traditional human–computer interaction recognition results and the measured results. Human computer interaction recognition mainly utilizes existing commercial image processing software to preprocess and binarize damaged area images, and then manually select specific areas, combined with the number of pixels, to calculate various parameter values. The measured value is mainly measured by Vernier scale for many times, and the average value of the five measured results is selected as the measured result. In terms of extraction results of corresponding length L(max) of largest damaged area in the bridge structure image, the comparison results are shown in Fig. 13. In terms of comparison of corresponding length of largest damage area, it can be seen from Fig. 13a that the results obtained by this method and the results obtained by traditional human–computer interaction are greater than the measured results. By using the measured values as the denominator and the interval value between the results obtained by this method and the measured values as the numerator, the calculated percentage value is considered as the result error of this method. By using the measured values as the denominator and the interval value between the results obtained by traditional human–computer interaction methods and the measured values as the numerator, the calculated percentage value is considered as the result error of traditional human–computer interaction methods. As can be seen from Fig. 13b, the maximum error of results obtained by this method is 1.931%, and the minimum error is 0.904%. The maximum error of results obtained by traditional human–computer interaction method is 47.967%, and the minimum error is 3.013%. The results show that the proposed method is more accurate in the extraction of corresponding length L(max) of largest damaged area in the bridge structure image.

**Figure 14:**
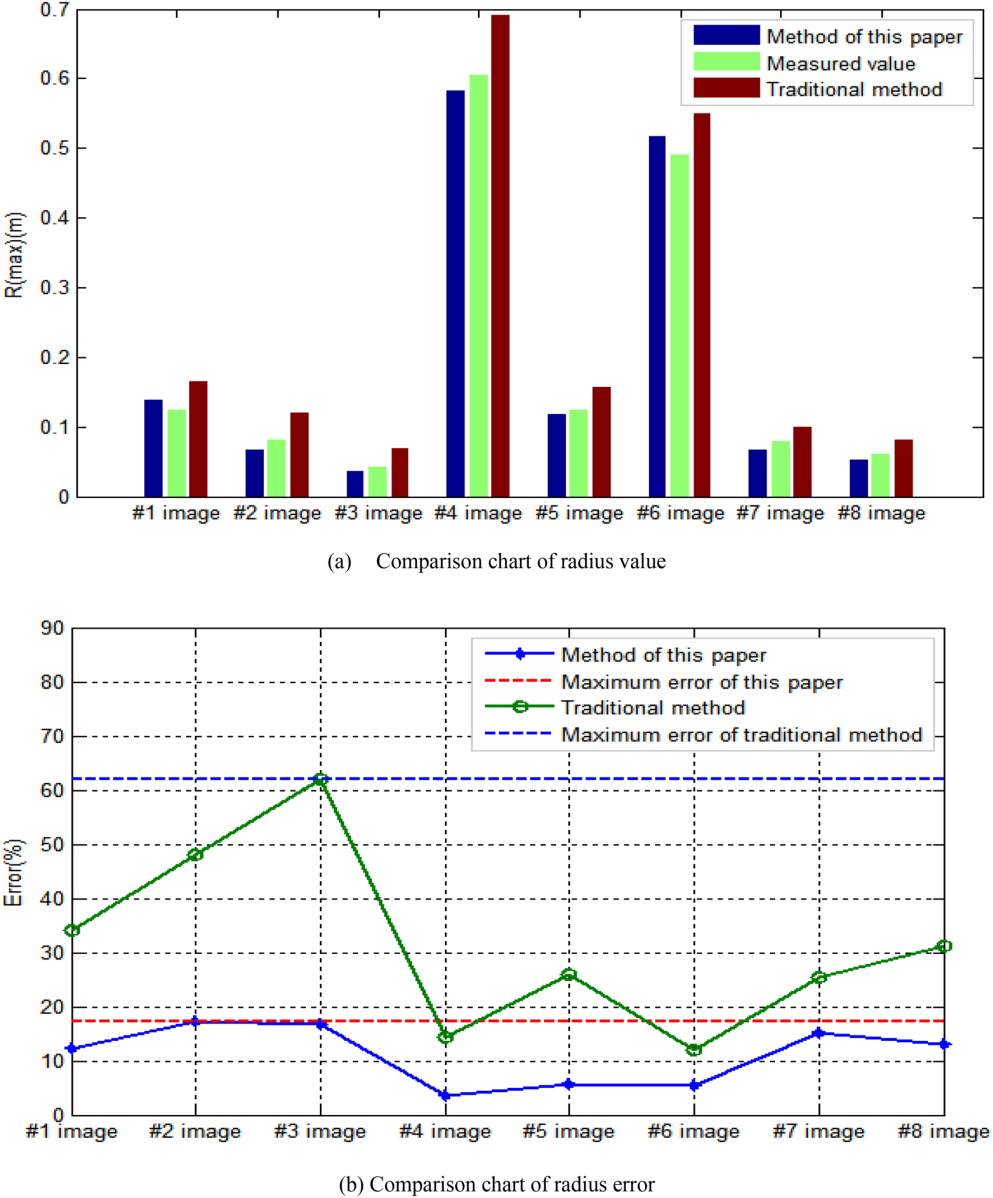
Comparison of radius value and error.

In terms of extraction results of radius R(max) corresponding to the largest damage area in the bridge structure image, the comparison results are shown in Fig. 14. As for the comparison of corresponding radius of maximum damage area, it can be seen from Fig. 14a that the results obtained by this method are closer to the measured results than those obtained by traditional human–computer interaction. As can be seen from Fig. 14b, the maximum error of results obtained by this method is 17.284%, and the minimum error is 3.477%. The maximum error of results obtained by traditional human–computer interaction method is 61.905%, and the minimum error is 12.016%. The results show that the proposed method is more accurate in the extraction of corresponding radius R(max) of largest damage area in the bridge structure image.

**Figure 15:**
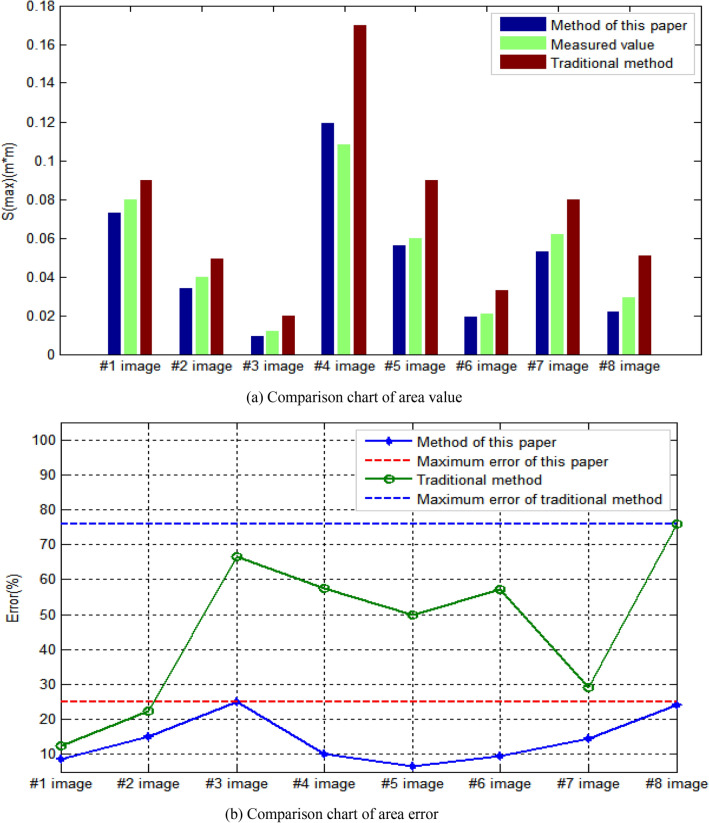
Area value and error comparison chart.

In terms of extraction results of corresponding area S(max) of largest damage area in the bridge structure image, the comparison results are shown in Fig. 15. As for the comparison of corresponding area of largest damage area, it can be seen from Fig. 15a that the results obtained by this method are closer to the measured results than those obtained by traditional human-computer interaction. By using the measured values as the denominator and the interval value between the results obtained by this method and the measured values as the numerator, the calculated percentage value is considered as the result error of this method. By using the measured values as the denominator and the interval value between the results obtained by traditional human-computer interaction methods and the measured values as the numerator, the calculated percentage value is considered as the result error of traditional human-computer interaction methods. As can be seen from Fig. 15b, the maximum error of results obtained by this method is 25.000 %, and the minimum error is 6.667 %. The maximum error of results obtained by traditional human-computer interaction method is 75.862 %, and the minimum error is 12.5 %. The results show that the proposed method is more accurate in the extraction of corresponding area S(max) of largest damage area in the bridge structure image.

**Figure 16:**
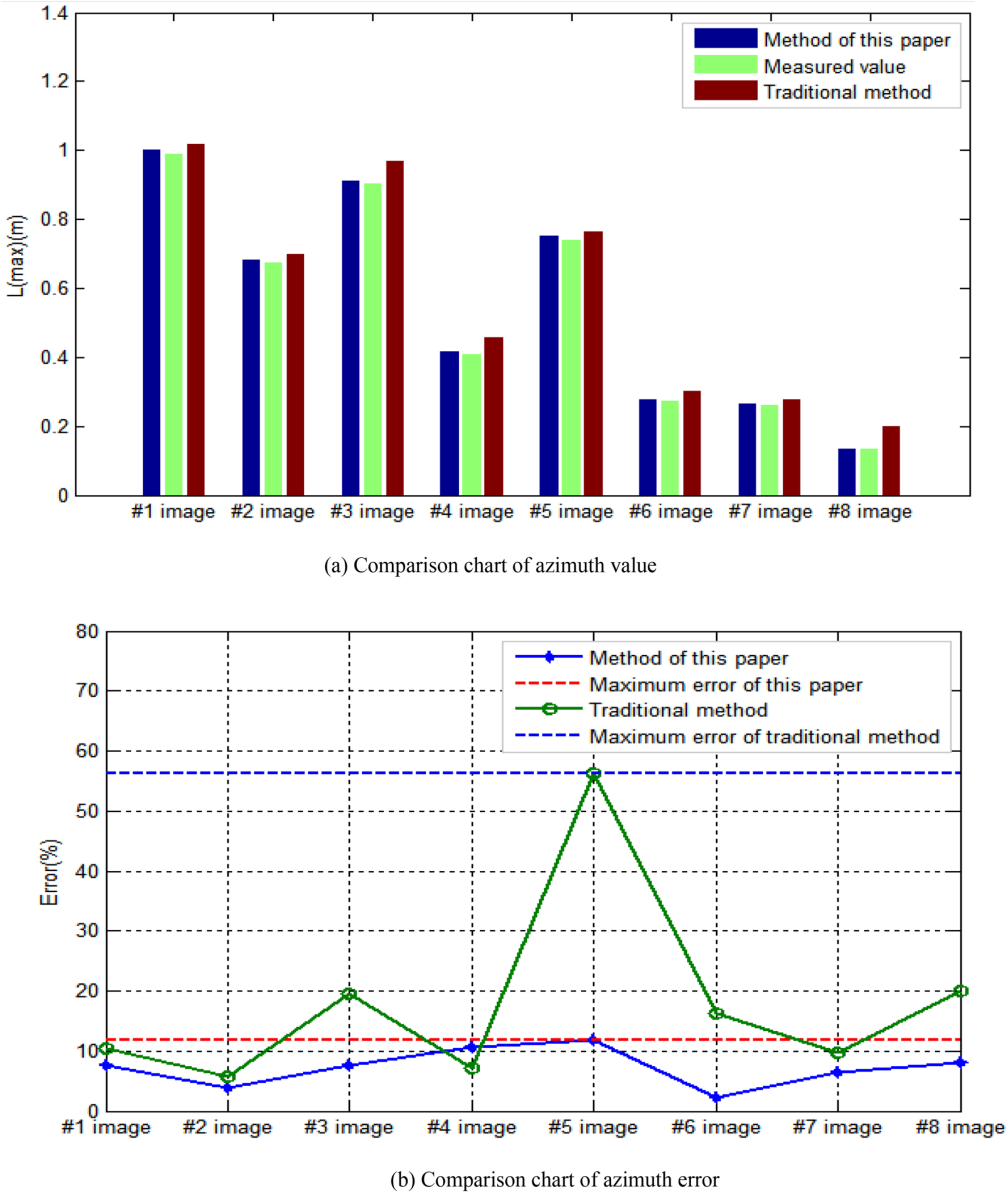
Comparison of azimuth value and error.

In the aspect of S_r_(max) extraction results of corresponding azimuth of largest damage area in the bridge structure image, the comparison results are shown in Fig. 16. As for the comparison of corresponding directions of largest damage area, it can be seen from Fig. 16a that the results obtained by this method are closer to the measured results than those obtained by traditional human–computer interaction. By using the measured values as the denominator and the interval value between the results obtained by this method and the measured values as the numerator, the calculated percentage value is considered as the result error of this method. By using the measured values as the denominator and the interval value between the results obtained by traditional human–computer interaction methods and the measured values as the numerator, the calculated percentage value is considered as the result error of traditional human–computer interaction methods. As can be seen from Fig. 16b, the maximum error of results obtained by this method is 11.844%, and the minimum error is 2.186%. The maximum error of results obtained by traditional human–computer interaction method is 56.250%, and the minimum error is 5.802%. It shows that the results obtained by this method are more accurate in the aspect of extracting the corresponding direction S_r_(max) of largest damage area in the bridge structure image.

In Figs. 13b, 14b, 15b, traditional methods were used to calculate length, radius, and area, but there were significant errors in # 3image and # 8 image. This was mainly due to the blurriness of # 3 image compared to other images and the presence of more small pores in # 8 image compared to other images. Blurred images and small-scale damage areas can easily lead to human recognition biases, thereby affecting machine recognition and computation. In Fig. 16b, when using traditional methods to calculate azimuth, there is a significant error in # 5 image. This is mainly due to the presence of more azimuth jitter in the damaged area of # 5 image, which does not have continuity, leading to the introduction of significant errors in calculating azimuth. Compared with traditional methods, the method proposed in this paper has more advantages in identifying areas of image blur, small-scale damage, and random damage.

By comparing eight groups of test results, it can be seen that using the method proposed in this article to extract the length of the damaged area has a measurement error of no more than 2%, the radius of the damaged area has a measurement error of no more than 18%, the area of the damaged area has a measurement error of no more than 25%, the azimuth of the damaged area has a measurement error of no more than 12%. It shows that the accuracy of this method is higher. This is mainly because in the image recognition of fracture features and cavity features, fracture features are easier to recognize. It can count and analyze the width and orientation of crack through the global pixel features, and is not easy to be affected by the local infected pixels. In addition, it can be seen that the accuracy of length, radius, area and orientation parameters of damage area extracted by this method is higher than that of traditional human–computer interaction results. This is mainly because the traditional human–computer exchange method needs manual intervention, and its calculation results are easily interfered by factors. The traditional human–computer interaction extraction has a great relationship with the experience of analyst,. The surface image of analyzed bridge structure is not preprocessed, which is easily affected by subjective judgment. This parameter extraction method can more objectively extract the characteristic parameters corresponding to the bridge structure damage area. By comparing the results of automatic extraction and traditional extraction, it can be seen that the feature parameters of damage area automatically identified in this paper are more accurate, and the overall error rate is lower than that of traditional extraction. In addition, the extraction efficiency of automatic identification in this paper is significantly higher than that of traditional human–computer interaction extraction method, which proves that the bridge structure based on digital image described in this paper. The method of damage area automatic identification is feasible and accurate. The crack scale applicable to this method is influenced by the comprehensive factors of image resolution, testing distance, and shooting angle. In general, the higher the resolution, the closer the testing distance, the more positive the shooting angle, and the smaller the scale of the identified cracks. This allows for more accurate collection and identification of the area and scale in which they are located. In an ideal situation, the crack recognition resolution can reach 0.1mm.

Because the method in this paper is to identify the structural damage area based on image recognition, there is no obvious requirement for the type of bridge structures, such as steel bridges and concrete bridges. Using digital camera can effectively capture clear images of bridge damage areas. Combined with this method, the parameters of the damaged area can be extracted, which is convenient for subsequent detection and analysis. In addition to the automatic identification of the damage area of bridge structures, this technique can also be applied to the damage detection of other conventional concrete structures and blocks. The method in this paper also has some shortcomings. Because the judgment of the depth direction of the damage area is more important to the overall quality evaluation, the data source used in this paper is the plane image of the damage area, which belongs to the two-dimensional information source, and it cannot effectively judge the depth of the damage area. Therefore, in order to understand the depth of the damage area of the bridge, it is also necessary to combine other nondestructive detection methods (such as acoustic wave and radar technology) for comprehensive analysis. In addition, the method described in this paper is mainly to extract the crack characteristics in the digital image, while in the actual bridge structure, some cracks are insignificant. Therefore, when making bridge structure detection and judgment, while using this technology, it is also necessary to combine the actual situation of the site to choose or reject some damage areas or cracks, ignoring some parameters that have no reference significance for detection and evaluation, so as to ensure the accuracy of bridge structure quality evaluation. According to the perspective correction process of the damaged area, it can be seen that the larger the angle between the plane of the bridge surface and the imaging plane, the lower reliability of the damage estimate. Therefore, in actual image acquisition, it is still necessary to minimize the angle as much as possible. A too large angle can also affect the correction effect of the method in this article.

## Conclusion

In this paper, the corresponding technical research is carried out to solve the technical problem of image recognition and measurement of bridge structure damage area. On the basis of defining the characteristics of the digital image of the damage area of the bridge structure, a preprocessing method of the image of the damage area of the structure is proposed, which can effectively improve the quality of the image taken on site. This method realizes the visual angle correction of the damage area and the enhancement of the damage feature area of the bridge structure surface image, and can effectively suppress the interference caused by black spots and dirty spots. In the automatic identification of the damage area of bridge structure, an improved Ostu method is proposed to organically fuse the global threshold and local threshold features of the image. The gradient difference feature is used to further filter the damage area to achieve the contour carving of the damage area of the bridge structure surface image, which can more accurately achieve the contour extraction of the damage area. In the quantification of the characteristic parameter index of the damage area of the bridge structure, the scale of the damage area, the proportion of the damage area and the orientation calculation rule of the damage area are constructed. The important inspection and characteristic parameter diagnosis of the damage area of the bridge structure are realized. Combined with the test analysis of actual engineering cases, the test results show that the automatic identification method of bridge structure damage area based on digital images proposed in this paper is feasible and stable, which can improve the measurement accuracy of the current bridge structure damage area, providing more data support for the detection and maintenance of bridge structures.

## Data Availability

All data generated or analysed during this study are included in this published article.
